# Frizzled-1 receptor regulates adult hippocampal neurogenesis

**DOI:** 10.1186/s13041-016-0209-3

**Published:** 2016-03-15

**Authors:** Muriel D. Mardones, Gabriela A. Andaur, Manuel Varas-Godoy, Jenny F. Henriquez, Felipe Salech, María Isabel Behrens, Andrés Couve, Nibaldo C. Inestrosa, Lorena Varela-Nallar

**Affiliations:** Center for Biomedical Research, Faculty of Biological Sciences and Faculty of Medicine, Universidad Andres Bello, Santiago, Chile; Centro de Investigación Biomédica, Facultad de Medicina, Universidad de los Andes, Santiago, Chile; Unidad de Geriatría, Hospital Clínico Universidad de Chile, Santiago, Chile; Instituto de Ciencias Biomédicas (ICBM), Facultad de Medicina, Universidad de Chile, Santiago, Chile; Departamento de Neurología y Neurocirugía, Hospital Clínico Universidad de Chile, Santiago, Chile; Clínica Alemana de Santiago, Santiago, Chile; Biomedical Neuroscience Institute (BNI), Santiago, Chile; Centro de Envejecimiento y Regeneración (CARE), Departamento de Biología Celular y Molecular, Facultad de Ciencias Biológicas, P. Universidad Católica de Chile, Santiago, Chile; Center for Healthy Brain Ageing, School of Psychiatry, Faculty of Medicine, University of New South Wales, Sydney, Australia; Centro de Excelencia en Biomedicina de Magallanes (CEBIMA), Universidad de Magallanes, Punta Arenas, Chile

**Keywords:** Adult neurogenesis, Hippocampus, Neural progenitor cells, Neuronal differentiation, Wnt signaling, Frizzled

## Abstract

**Background:**

In the adult hippocampus new neurons are continuously generated from neural stem cells (NSCs) present at the subgranular zone of the dentate gyrus. This process is controlled by Wnt signaling, which plays a complex role in regulating multiple steps of neurogenesis including maintenance, proliferation and differentiation of progenitor cells and the development of newborn neurons. Differential effects of Wnt signaling during progression of neurogenesis could be mediated by cell-type specific expression of Wnt receptors. Here we studied the potential role of Frizzled-1 (FZD1) receptor in adult hippocampal neurogenesis.

**Results:**

In the adult dentate gyrus, we determined that FZD1 is highly expressed in NSCs, neural progenitors and immature neurons. Accordingly, FZD1 is expressed in cultured adult hippocampal progenitors isolated from mouse brain. To evaluate the role of this receptor in vivo we targeted FZD1 in newborn cells using retroviral-mediated RNA interference. FZD1 knockdown resulted in a marked decrease in the differentiation of newborn cells into neurons and increased the generation of astrocytes, suggesting a regulatory role for the receptor in cell fate commitment. In addition, FZD1 knockdown induced an extended migration of adult-born neurons within the granule cell layer. However, no differences were observed in total dendritic length and dendritic arbor complexity between control and FZD1-deficient newborn neurons.

**Conclusions:**

Our results show that FZD1 regulates specific stages of adult hippocampal neurogenesis, being required for neuronal differentiation and positioning of newborn neurons into the granule cell layer, but not for morphological development of adult-born granule neurons.

## Background

The hippocampus is one of the two brain regions where neurogenesis takes place in the adult mammalian brain. In the dentate gyrus, radial glia-like neural stem cells (NSCs) that are present at the subgranular zone (SGZ), between the granule cell layer (GCL) and the hilus, divide and generate transient-amplifying progenitors that have the potential to differentiate into granule neurons that mature over several weeks and integrate into the hippocampal network [1, 2]. Adult generated neurons contribute to the plasticity of the hippocampus [3, 4] and are important for some forms of hippocampal-dependent learning and memory, including spatial memory, pattern separation and contextual fear conditioning [5–7].

The sequential steps of adult neurogenesis are tightly controlled by intrinsic and extrinsic factors. Progression of neurogenesis is coordinated by transcriptional and epigenetic mechanisms [8], local network activity [9], and signaling molecules including Wnts [10–13]. Wnt ligands are members of a family of 19 secreted glycoproteins in mammals that bind to seven-pass transmembrane Frizzled (FZD) receptors to trigger the activation of the canonical Wnt/β-catenin signaling pathway or the non-canonical Wnt/Ca^2+^ or Wnt/planar cell polarity (PCP) signaling cascades [14]. In vitro experiments in cultured adult hippocampal progenitors (AHPs) isolated from adult rat brain have shown that astrocyte-derived Wnts induce the differentiation of these progenitors into neurons [15]. On the contrary, endogenous Wnts produced by AHPs prevent differentiation and support proliferation and multipotency of progenitor cells, suggesting that autocrine Wnt signaling is relevant for the maintenance of NSCs [16]. In the SGZ of the adult rat hippocampus, the Wnt/β-catenin signaling pathway is active in proliferating cells [15]. Additionally, in vivo manipulation of Wnt activity indicated a role for Wnt signaling in cell proliferation and in the generation of new neurons [15]. In agreement with these observations, Wnt inhibitors Dickkopf 1 (Dkk1) and secreted frizzled-related protein 3 (sFRP3) negatively regulate neurogenesis under physiological conditions [17–19]. Moreover, sFRP3 deletion promotes dendritic development, dendritic spine formation and leads to accelerated maturation of newborn neurons [18], indicating that Wnt signaling is also relevant for maturation of adult-born neurons. Altogether, this evidence indicates that in the adult dentate gyrus Wnt signaling plays a complex role in regulating multiple steps of neurogenesis including proliferation of NSCs, differentiation of progenitor cells and development of adult-born neurons. Considering that all these steps of neurogenesis occur in the dentate gyrus where cells at different stages of neurogenesis are in close proximity, we proposed that cell-type specific activities of Wnts during this process might be determined by specific Wnt receptors [12]. In agreement, the expression of some Wnt receptors changes during differentiation in cultured AHPs [20]. During hippocampal development, FZD receptors also show differential expression [21], and in cultured hippocampal neurons FZDs show different cellular distributions that seems to correlate with their specific functions [21–24]. In the present study, we evaluated whether the receptor FZD1 contributes to hippocampal neurogenesis in the adult mouse brain. We specifically focused our study on FZD1 since it is expressed in the adult hippocampus [22, 25], is a well known receptor for the Wnt/β-catenin signaling pathway [25–29], and has been reported to act as a receptor for Wnt3 and Wnt3a [25, 26, 30, 31], which have been involved in adult neurogenesis [15, 32]. Furthermore, FZD1 has been shown to be a Wnt target gene that may function as part of a positive auto-feedback loop to control Wnt signaling [29, 31, 33]. We determined that FZD1 is expressed in NSCs, neural progenitor cells and immature neurons of the adult dentate gyrus. In vivo, FZD1 knockdown impaired neuronal differentiation and altered migration of newborn neurons within the GCL. However, FZD1 knockdown did not affect dendritic development. These findings indicate that FZD1 regulates specific steps of neurogenesis being important for neuronal differentiation and positioning of newborn neurons into the GCL.

## Results

### FZD1 is expressed in neural progenitor cells and immature neurons in the dentate gyrus of the adult hippocampus

**Fig. 1:**
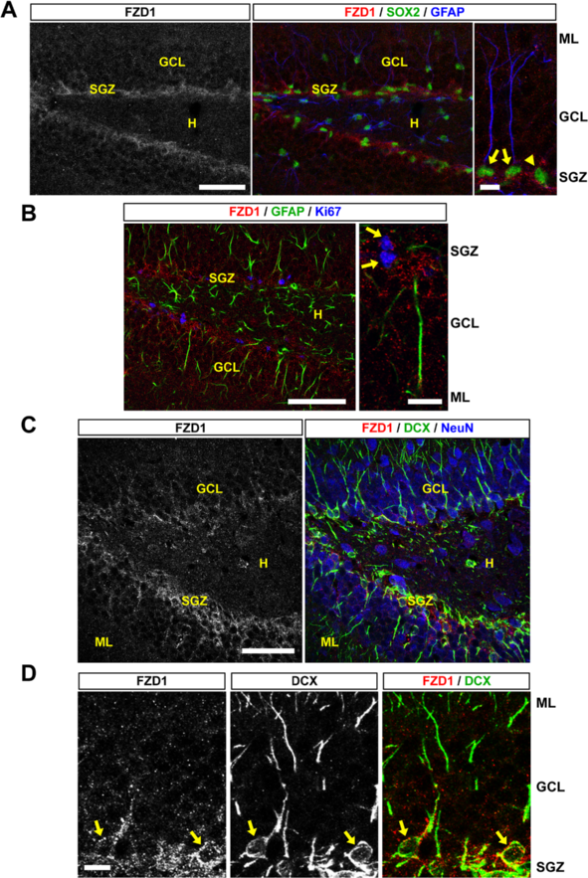
FZD1 is expressed in NSCs, amplifying progenitors and immature neurons of the adult mouse hippocampus. **a** Representative immunodetection of FZD1, SOX2 and GFAP in the dentate gyrus of 2-month-old mouse. Scale bar: 50 μm. Right, higher magnification of the image. *Arrows* indicate SOX2-positive cells with a single GFAP-positive projection. *Arrowhead* indicates a cell only positive for SOX2. Scale bar: 5 μm. **b** Immunodetection of FZD1, GFAP and the mitotic marker Ki67, scale bar: 50 μm. Right, higher magnification of the image. *Arrows* indicate cells positive for FZD1 and Ki67 staining. Scale bar: 10 μm. **c** Representative immunodetection of FZD1, DCX and NeuN in the dentate gyrus of 2-month-old mouse. Scale bar: 50 μm. **d** Higher magnification of the image shown in **c**. For simplification, only the double staining FZD1/DCX is shown. *Arrows* indicate FZD1-staining in the cell body of DCX-positive immature neurons. Scale bar: 10 μm. ML: molecular layer, GCL: granule cell layer, SGZ: subgranular zone, H: hilus

We first analyzed the expression of the FZD1 receptor in the dentate gyrus of 2-month-old mice by immunofluorescence. To evaluate whether the receptor is expressed in NSCs, FZD1-staining was evaluated in cells positive for glial fibrillary acidic protein (GFAP) and the transcription factor SOX2, two proteins present in radial glia-like NSCs in the SGZ [34]. FZD1-staining was observed in the GCL, being more prominent in the inner layer of the GCL and in the SGZ (Fig. 1a). FZD1-staining was observed in SOX2-positive cells with a single GFAP-positive projection (Fig. 1a, arrows), indicating that the receptor is present in NSCs, and was also observed in cells only positive for SOX2 (Fig. 1a, arrowhead), suggesting that the receptor is present in transient-amplifying progenitors, which do not express GFAP. In agreement, FZD1 staining was detected in cells expressing the mitotic marker Ki67 that were negative for GFAP (Fig. 1b). Also, FZD1 colocalized partially with cells positive for the immature neuron marker doublecortin (DCX) (Fig. 1c). FZD1 staining was mostly observed in the cell bodies of DCX-positive cells present in the GCL (Fig. 1d, arrows).

**Fig. 2:**
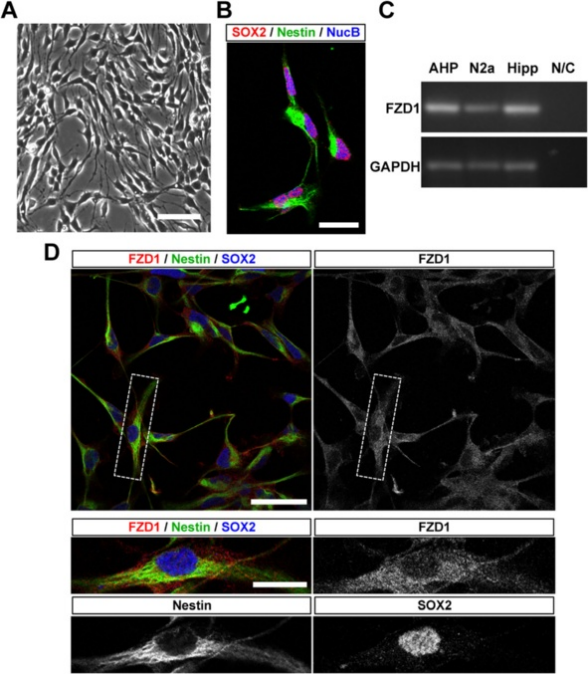
FZD1 is expressed in AHPs isolated from adult mouse hippocampus. **a** Monolayer of AHPs isolated from 6-week-old mouse hippocampus and cultured under proliferation conditions. Scale bar: 50 μm. **b** Immunoflurescence staining of SOX2 and nestin in AHPs. Nuclei were stained with NucBlue (NucB). Scale bar: 10 μm. **c** RT-PCR analysis of the expression of FZD1 and GAPDH in cultured AHPs (lane 1), N2a cells (lane 2), mouse hippocampus used as positive control (lane 3) and water used as PCR negative control (N/C, lane 4). **d** Immunoflurescence staining of FZD1, nestin and SOX2 in AHPs. Scale bar: 50 μm. *Bottom panels*, higher magnification of the cells indicated in top panels. Scale bar: 20 μm

The expression of FZD1 was also evaluated in cultured AHPs isolated from the adult mouse hippocampus [35]. AHPs showed characteristic progenitor cell morphology (Fig. 2a), and 93.00 ± 2.82 % were positive for SOX2 and the cytoskeletal protein nestin (Fig. 2b). RT-PCR analysis and immunofluorescence staining showed that FZD1 was expressed in cultured AHPs (Fig. 2c–d), supporting that FZD1 is expressed in neural progenitor cells of the adult dentate gyrus.

### FZD1 knockdown impairs neurogenesis in the adult dentate gyrus

**Fig. 3:**
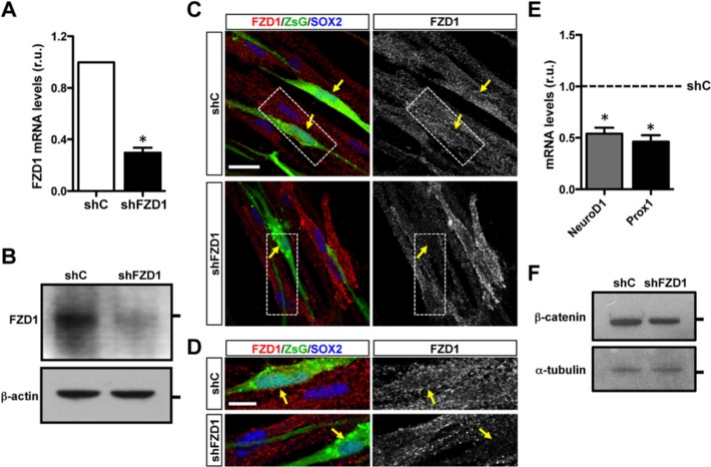
FZD1 knockdown in N2a cells and AHP isolated from adult mouse brain. **a** qRT-PCR from total RNA isolated from N2a cells 48 h after transfection with retroviral vectors expressing shRNAs (shC or shFZD1) and the fluorescent protein ZsGreen (ZsG). FZD1 mRNA levels were normalized to GAPDH mRNA and expressed relative to shC. *Bars* represent mean ± S.E. *p < 0.05, Student’s *t*-test (*N* = 7 independent experiments). **b** Immunoblot analysis of FZD1 and β-actin in total protein extracts from N2a cells 48 h after transfection with the retroviral vectors. *Lines* at the right indicate molecular weight standards: 70 kDa (*top*) and 40 kDa (*bottom*). **c** Immunofluorescence analysis of FZD1 and SOX2 in AHPs 96 h after transfection with retroviral vectors expressing shC or shFZD1. *Arrows* indicate transfected cells (ZsG+). Scale bar: 20 μm. **d** Higher magnification of the cells indicated by *dotted lines* in top panels. Scale bar: 20 μm. **e** qRT-PCR quantification of the mRNA levels of NeuroD1 and Prox1 in N2a cells 48 h after transfection with shC or shFZD1. mRNA levels were normalized to GAPDH and expressed relative to shC (*dotted line*). *Bars* represent mean ± S.E. *p < 0.05, Student’s *t*-test (*N* = 3 independent experiments). **f** Immunoblot analysis of β-catenin and α-tubulin used as loading control, in total protein extracts from AHP cells 48 h after transduction with retrovirus expressing shC or shFZD1. *Lines* at the right indicate molecular weight standards: 90 kDa (*top*) and 55 kDa (*bottom*)

Considering the expression of FZD1 in neural progenitors and newborn neurons, we analyzed the in vivo role of FZD1 in adult hippocampal neurogenesis. For this purpose we designed a mouse FZD1–targeting shRNA (shFZD1) that was cloned into a retroviral vector that co-expresses the fluorescent protein ZsGreen (ZsG). Firstly, the efficiency of shFZD1 was assessed in the mouse neuroblastoma cell line Neuro-2a (N2a), which expresses the FZD1 receptor (Fig. 2c). In N2a cells transfected with shFZD1 there was a significant knockdown of endogenous FZD1 compared to N2a cells transfected with a control shRNA (shC). Significant decreases were observed at the mRNA and protein levels as assessed by qRT-PCR (Fig. 3a) and immunoblot (Fig. 3b), respectively. Knockdown of FZD1 was also evaluated in cultured mouse AHPs transfected with the retroviral vectors (Fig. 3c). Reduced FZD1 staining was observed in AHPs transfected with shFZD1 (expressing ZsG, arrows) compared to neighboring non-transfected cells (not expressing ZsG) (Fig. 3d).

To evaluate the effect of FZD1 knockdown on Wnt/β-catenin signaling activity, N2a cells were transfected with shRNAs and the expression of the Wnt target genes Prox1 and NeuroD1 [32, 36] was evaluated by qRT-PCR. Cells transfected with shFZD1 showed decreased mRNA levels of both genes compared to N2a cells transfected with shC (Fig. 3e). In addition, β-catenin was reduced by 0.6-fold in AHPs transduced with the shFZD1-expressing retrovirus compared with cells expressing shC (Fig. 3f). These results suggest that FZD1-knockdown reduced the Wnt/β-catenin signaling.

**Fig. 4:**
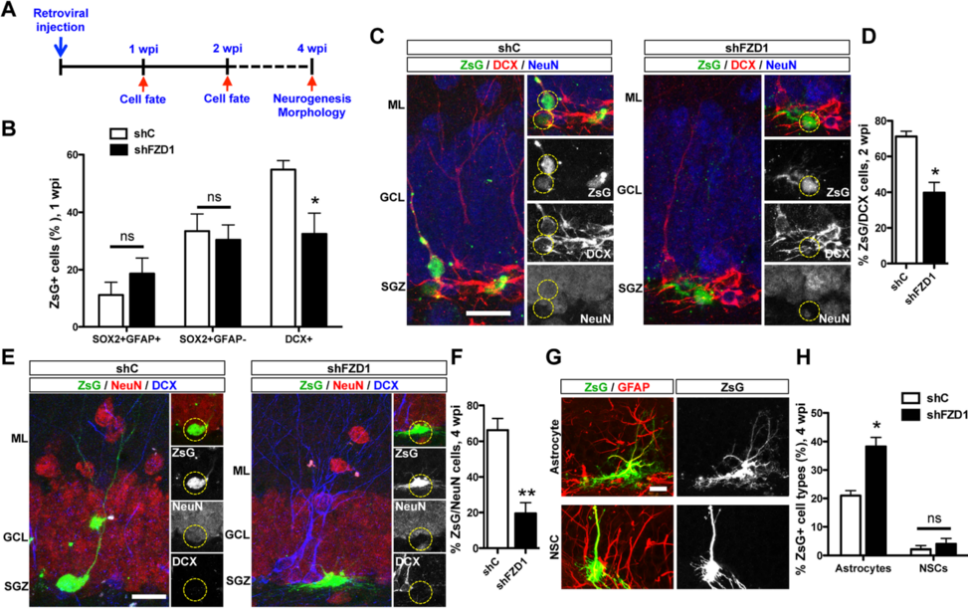
FZD1 knockdown reduces the generation of newborn granule neurons in the dentate gyrus. **a** Schematic representation of the experimental procedure. Retroviruses expressing shRNAs (shC or shFZD1) and ZsGreen (ZsG) were injected into the dentate gyrus of 2-month-old mice by stereotaxic surgery. Animals were sacrificed 1, 2 or 4 weeks post injection (wpi) to analyze neuronal fate commitment (1 and 2 wpi), generation and morphology of newborn granule neurons (4 wpi). **b** Quantification of the percentage of ZsG+ cells expressing SOX2/GFAP and the immature neuronal marker DCX 1 wpi. *Bars* represent mean ± S.E. *p < 0.05, Student’s *t*-test (*N* ≥ 4 mice). **c** Immunostaing of DCX and NeuN in brain sections from animals sacrificed 2 wpi. Images at the right show separated channels of a section of the images shown at the left. *Dotted circles* indicate transduced cells (ZsG+) in the merged images and in the separated channels. Scale bar: 20 μm. **d** Quantification of the percentage of ZsG+ cells expressing DCX at wpi. *Bars* represent mean ± S.E. *p < 0.05, Student’s *t*-test (*N* ≥ 3 mice). **e** Immunostaing of DCX and NeuN in brain sections from animals sacrificed 4 wpi. Images at the right show separated channels of a single z-plane section of the images shown at the left. *Dotted circles* indicate transduced cells (ZsG+) in the merged images and in each channel. Scale bar: 20 μm. **f** Quantification of the percentage of ZsG+ cells expressing the mature neuronal marker NeuN at 4 wpi. Bars represent mean ± S.E. *p < 0.01, Student’s *t*-test (*N* ≥ 3 mice). **g** Representative ZsG+ astrocytes and radial glia-like NSCs expressing GFAP. Scale bar: 20 μm. **h** Quantification of the percentage of ZsG+ cells that show astrocyte or NSCs morphology at 4 wpi. Bars represent mean ± S.E. *p < 0.01, Student’s *t*-test (*N* = 4 mice). ML, molecular layer; GCL, granule cell layer; SGZ, subgranular zone

Then, to knockdown FZD1 expression in proliferating cells in vivo shRNA-expressing retroviruses were stereotaxically injected into the dentate gyrus of 2-month-old mice. Mice were sacrificed 1 and 2 weeks post injection (wpi) to evaluate differentiation of newborn cells, and 4 wpi to evaluate neurogenesis and morphology of newborn granule neurons (Fig. 4a). Immunostaining of GFAP and SOX2 was carried out at 1 wpi to evaluate whether FZD1 knockdown affected the pool of NSCs and neural progenitor cells. No differences were observed in the percentage of ZsG-positive (ZsG+) cells SOX2 + GFAP+ and SOX2 + GFAP- between shC and shFZD1 mice (Fig. 4b). To evaluate neuronal differentiation, DCX staining was analyzed in control and FZD1-deficient cells. DCX is known to be transiently expressed in newly generated neuroblasts and immature neurons and its expression decreases as mature neuronal markers begin to be expressed [37]. A decrease in the percentage of newborn ZsG+ cells expressing DCX was observed 1 wpi in shFZD1 mice compared with mice injected with shC-expressing control viruses (Fig. 4b). At 2 wpi, ZsG+ cells were observed in the SGZ in mice injected with shC and shFZD1 expressing retroviruses (Fig. 4c). Most shC-expressing cells were positive for DCX staining (Fig. 4d), and as expected for 2-week-old neurons, ZsG+ cells were negative for the mature neuronal marker NeuN (Fig. 4c). On the contrary, most FZD1-deficient cells were negative for both DCX and NeuN (Fig. 4c). There was a significant reduction in the percentage of ZsG + DCX+ cells in shFZD1 mice compared with control animals (Fig. 4d). These results suggest that neuronal differentiation is impaired in FZD1-deficient cells.

To assess the effect of FZD1 knockdown on the generation of new granule neurons, we evaluated the expression of NeuN in ZsG+ cells at 4 wpi, which is when adult-born neurons should express this mature neuronal marker [37]. ZsG+ cells were observed in the dentate gyrus of mice injected with shC and shFZD1-expressing retroviruses (Fig. 4e); however, whereas most ZsG+ cells expressing shC expressed NeuN, most FZD1-deficient cells did not express this mature neuronal marker (Fig. 4f). There was a strong decrease in the percentage of ZsG + NeuN+ cells in shFZD1-expressing mice compared with control animals (Fig. 4f). Of note, ZsG + NeuN- cells did not express DCX either (Fig. 4e). In fact, no cells ZsG + DCX+ were observed at 4 wpi in mice injected with shC and shFZD1 retroviruses, indicating that ZsG + NeuN- cells were not neurons in an immature state. Also, we analyzed the expression of GFAP at 4 wpi, and determined that in mice injected with shFZD1 there were an increased percentage of ZsG+ cells expressing GFAP compared with control mice (shC: 23.06 ± 1.21; shFZD1: 42.23 ± 2.5 % of ZsG+ cells, *p* = 0.0139). Based on morphology of GFAP+ cells we quantified the percentage of radial glia-like NSCs and astrocytes (Fig. 4g), and determined that in shFZD1-injected mice there was a significant increase in the percentage of ZsG+ astrocytes (Fig. 4h), while there was no change in the percentage of radial glia-like NSCs. These results suggest that FZD1 knockdown impaired normal differentiation of newborn cells decreasing the percentage of cells that differentiate into granule neurons and increasing differentiation into astrocytes. Taken together these results demonstrate that FZD1 knockdown impairs neurogenesis in the adult hippocampus.

### FZD1 knockdown changes the migration but not dendritic arborization of adult-born granule neurons

**Fig. 5:**
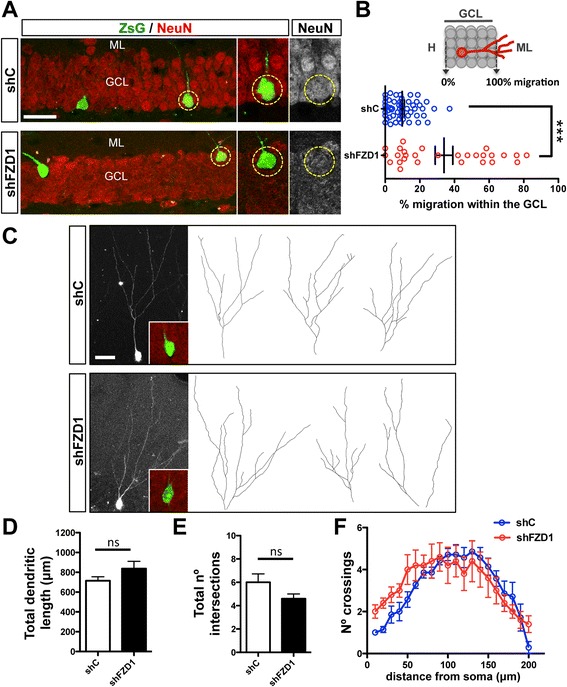
FZD1 knockdown affects migration but not dendritic development of newborn neurons. **a** Immunofluorescence staining of NeuN in animals sacrificed 4 wpi. Scale bar: 30 μm. *Right panels*, single focal planes of merged images (ZsG/NeuN) or NeuN staining separated. *Dotted circles* indicate the neuron that is shown in right panels. **b** The percentage of migration of ZsG + NeuN+ cells into the GCL was evaluated as shown in the sketch. The graph shows the migration of 4-week-old ZsG + NeuN+ cells. ***p < 0.001, Student’s *t*-test. **c** Denditric development of 4-week-old ZsG+ neurons. Images show 2D reconstructions of neurons infected with shC (t*op panel*) and shFZD1 (*bottom panel*). *Insets* show representative NeuN-staining in ZsG+ cells. **d**, **e** Total dendritic length (**d**), and total number of intersections (**e**) of 4-week-old ZsG+ neurons. *N* ≥5 neurons; ns: not significant, Student’s *t*-test, *p* = 0.141 (**d**), *p* = 0.163 (E). **f** Sholl analysis of dendritic complexity of ZsG + NeuN+ cells. ML, molecular layer; GCL, granule cell layer; H, hilus

To evaluate whether other steps of neurogenesis were affected by knockdown of FZD1, we analyzed the small percentage of ZsG+ cells that did express NeuN. In these cells, we evaluated migration into the GCL and dendrite development, both processes regulated by the Wnt signaling pathway in the adult hippocampus [18, 20]. It has been shown that adult-born neurons remain primarily positioned within the inner third of the GCL [38, 39]. As expected, at 4 wpi ZsG + NeuN+ cells expressing shC were located primarily within the first third of the GCL whereas shFZD1-expressing cells showed overextended migration (Fig. 5a and b). This result suggests that FZD1-mediated signaling regulates newborn neuron migration into the GCL in the adult dentate gyrus.

To evaluate dendritic arborization of newborn neurons (Fig. 5c), we selected all ZsG + NeuN+ cells (Fig. 5c, inset), with a complete ZsG+ dendritic arbor. No significant differences were observed in total dendritic length (Fig. 5d) or number of intersections (Fig. 5e) between newborn neurons expressing shC and shFZD1, suggesting that the FZD1 receptor is not required for dendrite development in adult-born granule neurons. To further analyze dendritic arborization, Sholl analysis was carried out in 4-week-old ZsG+ neurons, which revealed no significant differences in dendritic complexity between control and FZD1-deficient neurons (Fig. 5f).

## Discussion

The Wnt signaling pathway plays multiple roles during adult neurogenesis, regulating proliferation, differentiation and maturation of newborn neurons [15–18, 40]. Here we studied the potential role of the Wnt receptor FZD1 during adult hippocampal neurogenesis. We specifically focused on FZD1 since it is expressed in the adult hippocampus [22, 25], and is well known to activate the canonical Wnt/β-catenin signaling pathway [25–29]. We found that in the adult dentate gyrus, FZD1 is mainly expressed in NSCs, amplifying progenitors and immature neurons, suggesting that FZD1 may mediate the activation of the Wnt pathway in these cell-types. In agreement, the expression of FZD1 was also found in cultured AHPs that were isolated from hippocampi of adult mice.

The in vivo role of FZD1 in adult neurogenesis was evaluated by retrovirus-mediated shRNA knockdown of FZD1 expression in newborn cells of the dentate gyrus. We determined that differentiation into neurons was impaired in FZD1-deficient cells. In young mice the percentage of neuronal differentiation is normally 70–80 % [41–43], very similar to what was observed in mice expressing shC. Instead, we determined that ~40 % of newborn FZD1-deficient cells were positive for the immature neuron marker DCX at 1 and 2 wpi, suggesting that FZD1-mediated signaling is involved in neuronal differentiation during adult neurogenesis. Importantly, as determined by immunostaining for SOX2 and GFAP, no changes were observed in the pool of shRNA-expressing NSCs or neural progenitor cells between shC and shFZD1 mice, suggesting that the reduction in ZsG+ cells that became DCX+ neuroblasts and immature neurons was not due to a reduced progenitor cell population, but more likely due to impaired neuronal differentiation of progenitor cells. Moreover, 4 weeks after the retroviral injection there was a strong decrease in the percentage of FZD1-defcient cells that became granule neurons. Of note, FZD1-deficient cells that did not become granule neurons were not in an immature state, suggesting that the neuronal differentiation deficit is not a consequence of a delayed development of newborn neurons. Concomitantly with the decreased generation of new neurons, there was an increased percentage of FZD1-deficient cells that expressed GFAP and showed astrocyte morphology, supporting that FZD1-deficiency impaired normal differentiation. However, we cannot eliminate the possibility that reduced survival of newborn neurons may also contribute to the strong reduction in FZD1-deficient cells that became granule neurons.

FZD1 receptor mediates the activation of the canonical Wnt pathway by different Wnt ligands [25–29]; in agreement, we determined in N2a cells and AHPs that FZD1-knockdown reduced Wnt/β-catenin signaling activity. Therefore, it is possible that in the SGZ FZD1 mediates the activation of canonical Wnt signaling in progenitor cells to induce neural differentiation. In agreement, it is known from Wnt/β-catenin reporter mice that the canonical Wnt pathway is active in neural progenitor cells in the SGZ [44], and it was recently determined that knocking down the expression of LRP6, which is one of the co-receptors required for the activation of the Wnt/β-catenin signaling pathway, decreased neuronal fate determination of newborn cells [20]. The mechanism involved in the regulation of neuronal differentiation by Wnts involves the expression of proneural Wnt target genes [32, 36, 40], including the transcription factors NeuroD1, which stimulates neuronal differentiation and survival of neural progenitor cells in the adult dentate gyrus [32, 45] and Prox1, which is required for initial granule cell differentiation in the adult hippocampus [36]. Interestingly, we determined that both genes were downregulated by FZD1 knockdown. Therefore, it is possible that FZD1 regulates neuronal differentiation in the adult hippocampus by mediating the activation of the canonical Wnt/β-catenin signaling pathway and the expression of proneural genes.

Although most FZD1-deficient cells did not become granule neurons, there was a small proportion of cells that expressed the mature neuronal marker and showed neuronal morphology. We hypothesized that these cells were transduced with the shFZD1-expressing retrovirus (or expressed the shRNA) after the differentiation process was initiated. In these cells we evaluated migration and dendrite development, two processes regulated by Wnt signaling in the adult dentate gyrus [18, 20]. Adult-born neurons do not distribute randomly in the dentate gyrus, instead these remain primarily within the inner third of the GCL [38, 39]. In agreement with that, we observed that in control animals newborn neurons were located in the inner part of the GCL close to the SGZ, while FZD1-deficient newborn neurons reached outer layers. The mechanisms that prevent newborn neurons from migrating to the outer GCL are not fully understood. It was recently suggested that non-canonical Wnt/PCP signaling is involved in this process [20], but also alteration of adult-born neurons migration was observed in mice overexpressing GSK-3β [46], a key enzyme of the Wnt/β-catenin pathway that is inhibited upon activation of the signaling cascade, suggesting that canonical Wnt signaling may also be involved in this process. Moreover, knockdown of Disrupted-In-Schizophrenia 1 (DISC1), which inhibits GSK-3β and regulates neurogenesis through the Wnt/β-catenin signaling pathway [47], leads to overextended migration of adult-born granule neurons [48]. Therefore, it is possible that the effect observed in the migration of FZD1-deficient cells might be a consequence of impaired activation of the Wnt/β-catenin signaling pathway in newborn neurons as also suggested by the impaired neuronal differentiation. On the other hand, no significant effect was observed in dendrite morphogenesis by knockdown of FZD1. Wnt signaling regulates dendrite development in early cultures of hippocampal neurons, which involves non-canonical Wnt signaling components [23, 49]. β-catenin has also been involved in dendrite development, but this effect depends on the membrane cadherin/catenin adhesion complex and does not require Wnt/β-catenin-dependent transcription [50]. More recently, it was also demonstrated that the non-canonical Wnt/PCP pathway is involved in morphological maturation of adult-born neurons in the hippocampus, while LRP6 knockdown showed no effects on granule cell morphogenesis [20]. This data supports the notion that FZD1 activates canonical Wnt signaling in progenitor cells and newborn neurons to regulate specific steps of neurogenesis, however, further analyses are required to fully elucidate the signaling cascades involved.

It was recently shown that unlike FZD1, FZD3 is required for dendrite morphogenesis, but it is not involved in cell-fate determination [20]. Interestingly, FZD3 is not present in NSCs but is present in immature and mature neurons at the dentate gyrus. This evidence suggests that there is a cell-type specific expression of FZD receptors during adult hippocampal neurogenesis, which could mediate the multiple effects of Wnt signaling during this process [12]. We previously determined that FZD receptors have specific expression profiles during postnatal hippocampal development and have particular cellular distributions in cultured hippocampal neurons isolated from rat embryos [21], which are associated with different roles of Wnts in neurons including polarity, dendrite and axon morphogenesis and synapse formation [22–24, 51]. A similar regulation might occur during adult neurogenesis where NSCs and newborn neurons at different stages of maturity may express a different repertoire of Wnt receptors to mediate the different effects of Wnt signaling during the progression of neurogenesis.

## Conclusions

In the present study we determined that FZD1: (i) is expressed in NSCs, neural progenitor cells and immature neurons of the adult hippocampus; (ii) is involved in neuronal differentiation of hippocampal progenitor cells; (iii) regulates migration of newborn neurons into the GCL; (iv) is not required for dendritic development of adult-born granule cells. Altogether, these findings demonstrate for the first time that FZD1 is required for adult hippocampal neurogenesis.

## Methods

### Animals

Adult C57/BL6 mice were used for all experiments. All procedures involving experimentation on animals were approved by the Bioethical Committee of Universidad Andres Bello and were conducted in accordance with the guidelines of the National Fund for Scientific and Technological Research (FONDECYT-Chile). Mice had access to water and food ad libitum in a 12:12 h light/dark cycle.

### Perfusion and postfixation

Animals were anesthetized (100 μg ketamine + 10 μg xylazine in 10 μl saline/g), and then transcardially perfused with saline, followed by 4 % paraformaldehyde (PFA, Sigma-Aldrich) in 0.1 M PBS. Brains were removed and placed in 4 % PFA in PBS for 24 h at room temperature, dehydrated in 30 % sucrose and kept at 4 °C until analysis.

### Tissue sectioning

After dehydration, brains were sectioned on a cryostat (Leica Microsystems) and collected in ice-cold-PBS in multiwell dishes as previously described [52]. Tissue sections were sequentially collected in six sets of serial slices of 40 and 60 μm thickness, therefore, each set contained slices covering the entire length of the hippocampus and corresponds to a representative sampling of the whole hippocampus.

### Immunofluorescence

Immunodetection of neuronal markers was carried out as previously described [53, 54]. Primary antibodies used were: rabbit anti-doublecortin (Cell Signaling Technology Inc.), monoclonal anti-NeuN (Millipore), goat anti-FZD1 (R&D Systems), goat anti-FZD1 (LifeSpan Biosciences, Inc.), rabbit anti-SOX2 (Cell Signaling Technology Inc.), monoclonal anti-GFAP (Sigma-Aldrich), monoclonal anti-Nestin (Millipore), rabbit anti-Ki67 (Abcam). As secondary antibodies, Alexa (Molecular Probes) and DyLight (Abcam) conjugated antibodies were used. NucBlue (Life Technologies) was used as nuclear dye. Slices were mounted on gelatin-coated slides with Fluoromont-G (Electron Microscopy Sciences). Double-labeled sections were analyzed by confocal laser microscopy (Olympus FV 1000). Image analysis and z-projections were made with ImageJ software (NIH, USA).

### Isolation and culture of mouse AHPs

AHPs were isolated from the hippocampus of 6-week-old C57/BL6 mice and cultured in monolayers as previously described [35]. Cell suspension was plated in pre-treated plates coated with poly-D-lysine and laminin and cultured in proliferation medium consisting of Neurobasal A (Invitrogen) supplemented with B27 without vitamin A, Fungizone (Invitrogen), Glutamax (GIBCO), 100 U/ml penicillin and 100 μg/ml streptomycin and the growth factors FGF-2 (20 ng/mL, Alomone labs) and EGF (20 ng/mL, R&D systems).

### Transfection of AHPs and N2a cells

AHPs and N2a cells were transfected using Lipofectamine 2000 (Invitrogen) 2 days after plating in 24-well culture plates at a density of 20 × 10^4^ and 40 × 10^4^ cells per well respectively. Briefly, 0.5 μg of DNA and 0.75 μl of Lipofectamine 2000 were mixed in 100 μl Optimem (GIBCO). After 20 min the DNA-Lipofectamine 2000 reagent complex was added to the cells. For AHPs the media was replaced after 1 h at 37 °C with proliferation medium. For N2a cells the medium was replaced after 4 h for Dulbecco’s modified Eagle’s medium (GIBCO), supplemented with 10 % FBS (GIBCO), 100 U/ml penicillin and 100 μg/ml streptomycin. AHP and N2a cells were fixed with 4 % PFA, 4 % sucrose in PBS after 96 h or 48 h respectively.

### Immunoblot analysis

N2a cells were homogenized in RIPA buffer (10 mM Tris/HCl pH 7.4, 5 mM EDTA, 1 % NP-40, 1 % sodium deoxycholate and 1 % SDS) supplemented with a protease inhibitor mixture (1 mM PMSF, 2 μg/ml of aprotinin, 1 μg/ml of pepstatin and 10 μg/ml of benzamidine) and phosphatase inhibitors (25 mM NaF, 100 mM Na_3_VO_4_, 1 mM EDTA and 30 μM Na_4_P_2_0_7_). Homogenates were maintained on ice for 30 min and then centrifuged at 1000 g for 5 min (4 °C) to remove nuclei and large debris. Protein concentration on supernatants was determined using the BCA Protein Assay Kit (Pierce). Proteins were resolved in 10 % SDS/PAGE, transferred to a PVDF membrane and incubated overnight at 4 °C with primary antibodies. Primary antibodies used were: goat anti-FZD1 (R&D Systems), rabbit anti-β-actin (Cytoskeleton, Inc), mouse anti-β-catenin (Santa Cruz Biotechnology, Inc.), rabbit anti-α-tubulin (Santa Cruz Biotechnology, Inc.). The reactions were followed by incubation with peroxidase-conjugated secondary antibodies (Pierce) and developed using the ECL technique (Western Lightning Plus ECL, PerkinElmer).

### Reverse transcriptase and quantitative real-time PCR (qRT-PCR)

Total RNA was extracted using TRIzol reagent (Life Technologies) and reversely transcribed into complementary DNA (cDNA) using M-MuLV reverse transcriptase (New Englands BioLabs). qRT-PCR was performed using Brilliant II SYBR Green QPCR master mix (Agilent Technologies). Primers used were: FZD1: 5′-GGCCTGAAGATATGGAGTG-3′ (forward) and 5′-GGGGGAAGAAAGTAGGTTGC-3′ (reverse); Prox1: 5′ -CAGCGGACTCTCTAGCACAG-3′ (forward) and 5′ –GCCTGCCAAAAGGGGAAAGA-3′ (reverse); NeuroD1 5′ –CCTGATCTGGTCTCCTTCGTA-3′(forward) and 5′ –CAAGAAAGTCCGAGGGTTGA-3′ (reverse); GAPDH: 5′-CATGGCCTTCCGTGTTCCTA-3′ (forward) and 5′-CCTGCTTCACCACCTTCTTGAT-3′ (reverse). GAPDH: 5′-CATGGCCTTCCGTGTTCCTA-3′ (forward) and 5′-CCTGCTTCACCACCTTCTTGAT-3′ (reverse). mRNA levels were calculated using 2^ΔΔCT^ method and normalized to GAPDH gene.

### Retrovirus production and stereotaxic injection

To knockdown FZD1 an inverted and self-complementary hairpin DNA oligonucleotides encoding a short-hairpin RNA targeting mouse FZD1 mRNA were chemically synthesized (Life Technologies), aligned and cloned into the retroviral vector pSIREN-RetroQ (Clontech) that co-express the fluorescent protein ZsG. The control shRNA was provided by manufactures (Clontech). Oligos used to construct the shRNA targeting mouse FZD1 were: 5′-GATCCGGGTGGTGTGCAACGACAAGTTTTCAAGAGAAACTTGTCGTTGCACACCACCCTTTTTTACGCGTG-3′ (forward); 5′-AATTCACGCGTAAAAAAGGGTGGTGTGCAACGACAAGTTTCTCTTGAAAACTTGTCGTTGCACACCACCCG-3′ (reverse).

Retrovirus particles were prepared as previously described [55] with some modifications. HEK293T cells were plated 5 × 10^6^ cells per 100 mm plate for a total of ten plates, and after 24 h were co-transfected with 13.5 μg shRNA-expressing retroviral vectors, 9 μg packaging (pCMVgp) and 4.5 μg envelope (pCMV-VSV-G) vectors using polyethylenimine 18 mM pH 7. After 16 h medium was replaced by fresh medium. Retrovirus-containing supernatant was harvested 60 h post-transfection, centrifuged to eliminate cell debris, filtered through 0.45 μm cellulose acetate filters, and concentrated by two rounds of ultracentrifugation. Retroviral pellet was resuspended in PBS and aliquots were immediately stored at −80 °C. Titers were determined by transducing HEK293T cells with 3-fold serial dilutions of concentrated retrovirus and measuring the ZsG expression of infected cells 48 h after transduction.

For retrovirus injection into the dentate gyrus of 2-month-old mice, 1.5 μL of shRNA-expressing retroviruses were stereotaxically injected into the dentate gyrus using the following coordinates: 1.5 mm lateral; 2 mm anterioposterior; 2.3 mm ventral from bregma as previously described [55]. Retroviruses used in this study lack nuclear import mechanisms, thus viral integration occurs only in proliferating cells [56].

### Differentiation, migration and morphological analyses

To analyze neuronal differentiation of newborn cells, animals were sacrificed 2 or 4 weeks wpi Brain sections were processed for immunostaining and analyzed by confocal laser microscopy. z-stacks of ZsG-positive cells were carried out to analyze expression of neuronal markers. Images were acquired with a 60X objective and a z-axis interval of 1 μm. For quantification, all ZsG-positive cells in one set of tissue sections (see tissue sectioning) were analyzed. Image analysis and z-projections were made with ImageJ software (NIH). To analyze migration of newborn neurons, GCL thickness was estimated by using NeuN staining and considered as 100 %. Migration within the GCL at 4 wpi was evaluated for all ZsG + NeuN+ in one set of tissue sections (see tissue sectioning). For morphological analysis at 4 wpi, images of ZsG + NeuN+ were acquired with a 60X objective and a z-axis interval of 0.5 μm. Branching reconstruction, analysis of total dendritic length and number of intersections were made using the NeuronJ plugin. Sholl analysis was performed using the NIH ImageJ software with the Sholl analysis plug-in. Dendrite intersections were assessed at radial distances of 10 μm from the soma. For these analyses, all ZsG + NeuN+ cells in 3–4 sets of tissue sections were used.

### Statistical analyses

Statistical analyses were performed using Prism5 software (GraphPad Software Inc.). To compare averages between two groups the unpaired Student’s *t*-test was used. For Sholl analysis two-way ANOVA was used followed by Bonferroni post-test. In all graphs the data represent the mean ± SEM. Number of animals per experimental group or number of neurons analyzed in each experiment is indicated in Figure legends.

## Abbreviations

AHPs: adult hippocampal progenitors
DCX: doublecortin
DISC1: Disrupted-In-Schizophrenia 1
DKK1: Dickkopf 1
FZD: Frizzled
GCL: granule cell layer
GFAP: glial fibrillary acidic protein
N2a: neuro-2a
NSCs: neural stem cells
PCP: planar cell polarity
sFRP3: secreted frizzled-related protein 3
SGZ: subgranular zone
shC: control shRNA
shFZD1: FZD1-targeting shRNA
wpi: weeks post injection
ZsG: ZsGreen
